# Evaluation of the Effects of E-Learning on Nurses’ Behavior and Knowledge Regarding Venous Thromboembolism

**Published:** 2019-04

**Authors:** Fatemeh Bahrambeygi, Rahim Roozbahani, Davood Shojaeizadeh, Roya Sadeghi, Shamsi Nasiri, Elham Ghazanchaei, Shiva EhsanMaleki

**Affiliations:** 1 Department of Health Education and Promotion, School of Public Health, Tehran University of Medical Sciences, Tehran, Iran; 2 Clinical Tuberculosis and Epidemiology Research Center, National Research Institute of Tuberculosis and Lung Diseases (NRITLD), Shahid Beheshti University of Medical Sciences, Tehran, Iran; 3 School of Public Health, Tehran University of Medical Sciences, Tehran, Iran; 4 Chronic Respiratory Diseases Research center, NRITLD, Shahid Beheshti University of Medical Sciences, Tehran, Iran; 5 Lung Transplantation Research Center, NRITLD, Shahid Beheshti University of Medical Sciences, Tehran, Iran

**Keywords:** E-learning, Nurse, Knowledge, Behavior, Education, Traditional

## Abstract

**Background:**

Continuing medical education (CME) is an integral part of nursing professionalization, which can be effective in the development of nursing behavior. E-learning can play an important role in CME programs. This study aimed to evaluate the effectiveness of an E-learning program in increasing the nurses’ knowledge and behavior regarding care for venous thromboembolism (VTE) patients.

**Materials and Methods:**

One-hundred nurses were selected via convenience sampling method and divided into E-learning and traditional education groups. All nurses had access to the Internet, as well as adequate Internet literacy. Each nurse in the E-learning group received three didactic files during the intervention (four weeks), which focused on the VTE risk assessment, methods of prophylaxis, prophylaxis guidelines, diagnosis, pharmacological and non-pharmacological treatments, and patient education. On the other hand, nurses in the traditional education group were taught traditionally by an expert lecturer, who used audiovisual materials for teaching. A pretest-posttest analysis and a checklist were used to evaluate the impact of interventions in the groups. Chi-square and Mann-Whitney tests were also used to analyze the data.

**Results:**

The comparison of knowledge level between the groups showed that E-learning is not superior to traditional learning methods. The mean changes in the nurses’ behavior was 3.16±1.49 in the E-learning group and 2.77±1.26 in the conventional education group. Statistical analysis showed a significant relationship between the score changes and E-learning.

**Conclusion:**

Integration of E-learning in CME programs, besides attendance of traditional courses, can be an effective learning method. We suggest that future studies compare the effects of these methods.

## INTRODUCTION

Continuing medical education (CME) is the process through which health professionals keep themselves updated to meet the patients’ needs, provide health services, and facilitate their own professional development. It includes continuous acquisition of new knowledge, skills, and behaviors to enable competent practice (1). Rapid scientific advances force healthcare professionals to update their knowledge constantly. Due to the widespread application of CME, selection of an appropriate teaching method is clearly linked to technological developments, overpopulation, economic problems, geographical distribution, demands for higher education, and people’s willingness for self-motivation and self-learning.

CME, based on needs assessment, can improve the learning outcomes (2). It is an essential part of nursing professionalization and can be helpful in the nursing practice development. Studies have shown that web-based teaching is as effective as conventional teaching methods in CME for nurses (3). It seems that CME programs are the most important area of E-learning due to several reasons. First, the population requiring CME is distributed widely in different geographical regions. Second, not all people in the community have the same amount of time for learning. Third, people’s educational needs can be different, depending on their geographical and occupational status. Finally, people’s learning interests may vary (4).

E-learning has been adopted in nursing as an appropriate alternative to traditional learning (5). Generally, E-learning is considered an inexpensive and effective approach, which can overcome many problems, such as shift work exhaustion, low motivation, family involvement, lack of replacement employees, and interference of CME programs with work (6). E-learning programs are also suitable for nurses, as they can resolve problems, resulting from the nurses’ inability to attend traditional courses because of shift schedules. In this comparative study, we aimed to compare E-learning with traditional education methods for the development of nurses’ knowledge and behavior regarding venous thromboembolism (VTE).

E-learning, as an emerging phenomenon, can overcome some of the traditional education barriers and provide accessible education and flexible learning. It is widely accepted that advances in information technology (IT) and learning methods have provided opportunities to create interactive, affordable, efficient, flexible, and learner-centered E-learning environments (7). E-learning, as a positive reaction to IT challenges in the academic setting, is characterized as follows: 1) Separation of time and/or space between the teacher and students, between the students themselves, and between the students and educational resources; 2) interactions between the teacher and students, between the students, and between the students and educational resources by means of one or more media, especially the Internet; and 3) a process of teaching and learning, not limited to the immediate time and/or place (8).

E-learning has been promoted as a cost-effective and convenient method, facilitating lifelong learning. It has several advantages over traditional learning, especially by enabling learning anytime and anywhere. In this method, students have access to online materials, regardless of time and place. They can also reflect on the learning materials and their responses and work at their own pace, regardless of their race, sex, disability, or appearance (9). Moreover, E-learning offers opportunities for students to preview and practice the materials. Studies also suggest that students are highly satisfied with online learning (10). Moreover, the multi-session use of online resources may lead to the reinforcement of E-learning (11).

While E-learning has many advantages, it is important to consider its disadvantages, as well, which include little or no in-person contact with faculty members, feeling of isolation, steep learning curves of navigation systems, problems with technology use, need for students’ active involvement in the learning process, and increased lead time required for feedback to the assignments (12). In this regard, a review study by Cook et al. demonstrated that use of E-learning or Internet-based education has positive effects on the knowledge, skills, and behaviors of healthcare professionals, as well as patient outcomes (13). Also, a recent study (14) revealed that online learning has many benefits for busy professionals, particularly due to the ease of access and use.

In the past decade, many studies have been published, indicating the impact of information and communication technologies (ICT) on nursing education. Moule et al. demonstrated that nurses have positive attitudes toward online learning and suggested its various benefits for busy professionals (15). Moreover, Morente et al. reported the suitability of E-learning as an effective instrument for teaching the assessment and treatment of pressure ulcers and confirmed its potential impact on the clinical decision-making of nurses (16). Also, Khatoni et al. compared the effects of web-based and traditional education on nurses’ knowledge about avian influenza in a CME program (17). They found that web-based teaching is as effective as traditional teaching in nursing CME programs.

Moreover, a review study by Lahti et al. compared the impact of E-learning with traditional education on the acquisition of knowledge, skills, and satisfaction among nurses (18). However, their results showed that there was no significant difference between E-learning and traditional learning. Also, Lee et al. suggested that use of an E-learning program for pediatric medication management is an effective learning method, besides attendance of a standard lecture course (5). This type of E-learning seems to be suitable for pediatric nursing courses with limited class hours and can help students deal with unfamiliar situations.

Al-Hameed et al. recommended a CME educational program to improve the use of VTE prophylaxis (19). Their study revealed that appropriate VTE prophylaxis for patients, who developed VTE during hospitalization, increased significantly after a hospital-wide CME program, and the rate of hospital deaths associated with VTE tended to decrease after the CME program. Similar biomedicine studies have also shown the advantages of E-learning systems in professional educational programs (5, 16, 20).

Comparison of the knowledge and skills of physicians before and after attending traditional and electronic CME courses showed that E-learning is an effective approach for deep learning (21). Moreover, a more recent study on the use of an online E-learning system for nurses in delirium care showed that an E-learning course on delirium had significant effects on the nursing staff’s delirium care for older patients. This study found a significant increase in the knowledge of delirium after nurses completed the E-learning program (22).

Generally, it can be difficult to increase the nurses’ motivation and participation in their own education and skill development. The five most important factors, preventing nurses from participation in CME programs, include work commitments, domestic responsibilities, time constraints, scheduling of CME activities, and cost of the programs, as confirmed in previous studies (23). E-learning is a powerful tool, which can overcome these obstacles and help nurses become active and responsible contributors to their own education.

With this background in mind, this study aimed to determine and compare the knowledge and behavior of nurses in Masih Daneshvari Hospital, Tehran, Iran, before and after participation in E-learning and traditional programs on VTE.

## MATERIALS AND METHODS

This comparative study was conducted at the National Research Institute of Tuberculosis and Lung Diseases of Masih Daneshvari Hospital, affiliated to Shahid Beheshti University of Medical Sciences, Tehran, Iran. The interventions included an E-learning program and a traditional course on VTE for the hospital nursing staff. According to our pilot study and the mean comparison formula, a sample size of 48 subjects per group was measured. Considering a 10% sample attrition, a total of 53 nurses were enrolled in each group. The inclusion criteria were as follows: 1) nurses with a bachelor’s degree or higher; 2) no history of participation in an E-learning or CME program on VTE; 3) willingness to participate in the study; 4) access to the Internet; and 5) adequate Internet literacy. On the other hand, the exclusion criteria were lack of participation in the educational sessions for the traditional education group and failure to receive E-mail messages for the E-learning group.

Random available sampling was used to select the samples. The subjects had the right to withdraw from the study at any time. The hospital wards were located in four separate buildings. Fifty-three nurses were selected randomly from the wards in the northern area of the hospital for the traditional education group, and 53 nurses were randomly selected from the wards in the southern area of the hospital for the E-learning group. At the beginning of the study, each group consisted of 53 nurses. However, during the study, three nurses from the traditional group and three nurses from the E-learning group were excluded due to lack of participation in the educational sessions and absence from the posttest.

A researcher-made questionnaire (pretest/posttest) and a checklist were used to evaluate the impact of the interventions. The questionnaire was designed in three sections. In the first section, demographic and occupational information was collected (eight questions), and in the second section, access and use of the Internet and E-mail and participation in the CME program were evaluated (12 questions). The third section of the questionnaire consisted of 20 questions, related to VTE, according to the available scientific resources. Also, knowledge related to VTE risk assessment, methods of prophylaxis, prophylaxis guidelines, diagnosis, pharmacological and nonpharmacological treatments, and patient education was examined. One point was assigned for each correct answer.

The content validity method was used to determine the validity of the tools. Relevant books, journals, and articles were studied, and the questionnaires were adjusted based on the opinions of the supervisors, statisticians, and consultants. To determine the content validity, the questionnaire was sent to ten cardiologists and pulmonary specialists, and their comments were taken into account. The test-retest method was used to determine the reliability of the questions. For this purpose, 20 nurses, with the same characteristics and conditions as the subjects under study, completed the questionnaire. Two weeks later, the questionnaire was re-submitted to the respondents, and the correlation coefficient was calculated. Also, the content validity index of the questionnaire was above 0.80, indicating its acceptable content validity. Moreover, the correlation coefficient for the questionnaire was 0.838, indicating its acceptable reliability.

Additionally, a checklist of 14 items, including behaviors related to VTE risk assessment, methods of VTE prophylaxis, prophylaxis guidelines, diagnosis, pharmacological and non-pharmacological treatments, and patient education, was designed for the study. One point was assigned for each correct behavior. The educational content, which was identical for both groups, was selected by studying the reference books and guidelines of the American Heart Association. To determine the validity of the checklist, it was sent to ten cardiologists and pulmonary specialists, and their comments were taken into account. The educational content focused on VTE risk assessment, methods of VTE prophylaxis, prophylaxis guidelines, diagnosis, pharmacological and nonpharmacological treatments, and patient education.

The educational content for the E-learning group included text files and color images. For the traditional education group, a similar educational content was developed, using the same images in Microsoft PowerPoint. The intervention proceeded by holding a meeting with the nursing staff in the E-learning group to inform them of the study objectives and ensure that they could use the Internet and email. The researcher was given the email addresses of all subjects with their satisfaction. The researcher also gave her email address and telephone number to all of the participants. All nurses in the E-learning group could contact the researcher via phone call or email to ask questions about the educational content.

Before the educational intervention, a written pretest was presented to the nurses for evaluating their background knowledge on VTE. Each nurse in the E-learning group received three didactic files during the study (four weeks). To remind the nurses to read their emails, they received regular text messages, containing an overview of the key messages related to VTE prevention and treatment. One day after the end of the intervention, a written posttest was presented to the nurses.

On the other hand, nurses in the traditional education group were taught traditionally by the training supervisor of Masih Daneshvari Hospital, who is a CME instructor at Shahid Beheshti University. The subjects and the instructor met in a classroom, equipped with standard audiovisual media. Before the class, the nurses completed the same pretest as the experimental group. Next, an instructor taught the educational content, developed in PowerPoint. The traditional class continued for four hours. After finishing the class, the nurses completed the posttest, which was the same as the posttest in the experimental group.

Moreover, the designed checklist was used to evaluate the nurses’ behaviors before and after the intervention in both groups. Before starting the educational intervention, the nurses’ behavior and attitude toward VTE were observed carefully to evaluate their background practice regarding VTE. One month after the intervention, their behaviors were evaluated using the same checklist. To encourage the nurses those who obtained scores above 15 for knowledge, and above 10 for behavior, received eight CME credits.

### Data analysis

All statistical analyses were performed in IBM SPSS Version 19.0. Shapiro-Wilk test was used to analyze the distribution of data. Since both knowledge and behavior data were not normally distributed, non-parametric Mann-Whitney and Kruskal-Wallis tests were performed to evaluate differences between the samples. The level of statistical significance was set at α=0.05 for all analyses.

## RESULTS

### Characteristics of the study sample

One-hundred nurses participated in the CME program. As can be seen in Table 1, most participants were female nurses (n=94, 94%). Almost half of the nurses were 30–40 years old (n=51, 51%), and more than half of them were married (n=52, 52%). Most participants had a bachelor’s degree in nursing (n=94, 94%), and nearly half of them were working both day and night shifts (n=41, 41%). The mean and standard deviation (SD) of working years as a nurse was 10.96±6.03 years. Differences between the groups were examined using Chi-square test, and it was found that the E-learning and traditional education groups were similar in terms of all variables.

**Table 1.** Descriptive statistics of the participants, characteristic variables for both groups Changes in knowledge and behavior.

	**E- Learning**				**Traditional**			
	**Knowledge**		**Behavior**		**Knowledge**		**Behavior**	
	**Before**	**After**	**Before**	**After**	**Before**	**After**	**Before**	**After**
**Gender**								
** Female**	10.89±2.53	16.70±1.98	3.64±0.81	6.89±1.57	10.57±2.09	14.91±1.53	3.54±0.62	5.91±1.11
** Male**	12.67±2.30	15.33±1.15	3.67±1.15	6.00±1.73	9.75±3.59	15.00±1.41	3.00±0.81	5.75±1.50
**Marital Status**								
** Single**	10.70±2.36	16.78±2.19	3.57±0.94	7.00±1.70	10.56±2.12	15.32±1.06	3.44±0.71	5.96±1.17
** Married**	11.26±2.69	16.48±1.78	3.70±0.72	6.70±1.48	10.44±2.32	14.52±1.78	3.56±0.58	5.84±1.10
**Educational Level**								
** Bachelor**	10.94±2.59	16.60±2.01	3.62±0.84	6.77±1.56	10.49±2.21	14.93±1.46	3.47±0.65	5.87±1.15
** Master**	12.00±1.00	17.00±1.00	4.00±0.00	8.00±1.73	10.67±2.51	14.66±2.51	4.00±0.00	6.43±0.57
**Shifts**								
** Morning**	10.77±2.61	17.00±1.73	3.77±0.72	7.15±1.21	10.00±2.34	14.55±1.50	3.78±0.44	6.22±0.97
** Morning and evening**	10.90±2.64	16.80±2.25	3.40±0.69	6.80±1.87	10.57±2.06	14.75±1.74	3.36±1.63	5.50±1.01
** Evening and night**	10.95±2.70	16.35±1.98	3.65±0.98	6.65±1.66	10.95±2.16	15.40±1.42	3.55±0.68	6.05±1.09
** Circulate**	11.71±2.13	16.43±2.22	3.71±0.75	6.86±1.77	9.33±2.58	14.50±1.04	3.17±0.75	5.67±1.63

**Figure 1.**
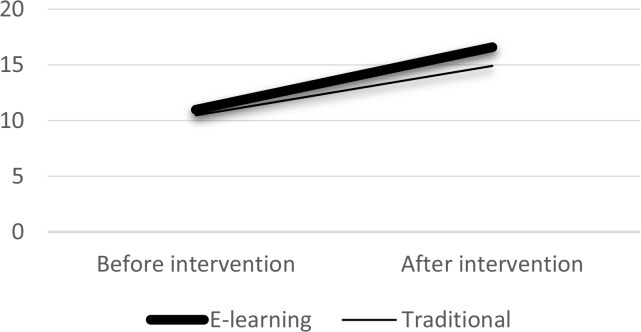
Changes in nurses’ knowledge for both groups

Before the E-learning program, the nurses had limited knowledge about VTE (11±2.53 out of 20). However, after the program, the average knowledge score of the E-learning group increased from 11±2.53 in the pretest to 16.62±1.96 in the posttest. On the other hand, in the traditional education group, the mean score of knowledge increased from 10.50±2.20 in the pretest to 14.92±1.50 in the posttest. The mean change of knowledge score was 5.62±2.85 in the E-learning group and 4.42±1.31 in the traditional education group. Although it seems that the mean change in the knowledge of the E-learning group was higher, the statistical analysis did not show any significant relationship between the score change and type of education (Figure 1).

### Changes in behavior

**Figure 2.**
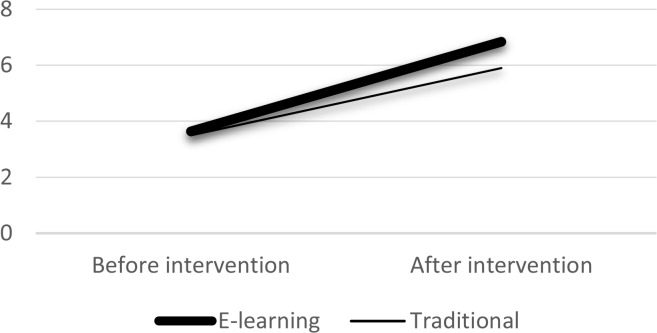
Changes in nurses ‘behavior in both groups

The results showed that the mean behavior score of the E-learning group increased from 3.64±0.82 before the program to 6.84±1.58 after the program. On the other hand, in the traditional education group, the mean score increased from 3.50±0.64 before the program to 5.90±1.12 after the program (behavior score was calculated out of 20) (Table 2). The mean change of behavior score was 3.16±1.49 in the E-learning group and 2.77±1.26 in the traditional education group. The statistical analysis showed a significant relationship between the score change and type of education. Overall, changes in the behavior of the E-learning group were greater than the changes in the traditional education group (Figure 2).

**Table 2.** Changes in knowledge and behavior.

	**E- Learning**		**Traditional**	

	**Before**	**After**	**Before**	**After**
**Knowledge**	11±2.53	16.62±1.96	10.50±2.20	14.92±1.50
**Behavior**	3.64±0.82	6.84±1.58	3.50±0.64	5.90±1.12

## DISCUSSION

This study aimed to investigate and compare the impact of E-learning and traditional education on the knowledge and behavior of nurses. The results of this study regarding the comparison of knowledge level showed that E-learning is not superior to traditional learning methods. Although the pretest results in both groups showed an increase in the level of knowledge compared to the posttest, there was no significant relationship between the educational methods and changes in the level of knowledge.

Also, we found that E-learning is more effective than traditional methods in improving the learners’ behavior. This finding is consistent with a randomized controlled trial by Hugenholtz et al., which showed that E-learning is as effective as lecture-based learning in enhancing knowledge (24). The present results are also in line with a recent review by Lahti et al., which indicated no significant difference between the E-learning and traditional education groups regarding the knowledge of nurses or nursing students (18).

Many studies have acknowledged that E-learning programs are flexible in time, location, and content (4, 25, 26). The results of the present study are consistent with a study by Lee et al. on 349 undergraduate nursing students, which showed that the E-learning group had greater improvements in pediatric medication management and learned how to integrate their medication knowledge and skills in practice (5). On the other hand, to improve the working nurse’s abilities by participating in CME programs, hospitals are increasingly cooperating with universities to provide educational opportunities on site and offer online courses for the students; therefore, E-learning can be both effective and cost-effective (27).

Several factors in the present study might have contributed to the improvement of learners’ behavior in E-learning. First, online CME learners completed the learning activities over several sessions, whereas traditional learners participated in a single session. Second, learners could check their emails several times; therefore, repeated checking of emails might have reinforced their learning. Third, the learners’ performance might have improved, since online CME learners could manage their learning process. The E-learning group could also use other sources on the Internet for expanding their knowledge, allocate time to each educational section as desired, and choose the place and time for learning. On the other hand, some nurses in the traditional learning group participated in the training class after their night shift; therefore, there is an obvious contrast between the E-learning and traditional learning groups (4, 18, 26). Also, online CME learners received regular text messages, containing an overview of the key messages of the session. These messages, as well as the multi-session use of online resources, might have led to learning reinforcement in E-learning (11).

### Limitations

This study had some limitations. First, since we implemented the first E-learning program in Masih Daneshvari Hospital, and the technological infrastructure was not advanced enough, the nurses had some problems using the Internet. Second, the short duration of the follow-up (one month) was not adequate to obtain reliable results. Finally, analysis of nurses’ skills and performance was limited due to time constraints.

## CONCLUSION

This interventional study compared E-learning with traditional learning methods for developing the nurses’ knowledge and behavior in caring for VTE patients. Based on the results, integration of E-learning in CME programs, besides attendance of traditional educational programs, is an effective learning method. Overall, the developed E-learning program was considered appropriate for resolving the nurses’ problems, resulting from their inability to attend traditional educational courses because of their tight work schedules. It is recommended to conduct further studies to compare retention in these teaching methods.
